# Impact of Exercise-Based Pulmonary Rehabilitation on Systemic Inflammation in Chronic Obstructive Pulmonary Disease (COPD): A Narrative Review on Evidence and Insights

**DOI:** 10.7759/cureus.110214

**Published:** 2026-06-03

**Authors:** Shivam Varfa, Astha Jindal, Priti Kochar, Anissa A Mirza, K. Ankush Patil, Upendra Panday, Pranjal Jain

**Affiliations:** 1 Pulmonary Medicine, All India Institute of Medical Sciences, Rishikesh, Rishikesh, IND; 2 Physiology, All India Institute of Medical Sciences, Rishikesh, Rishikesh, IND; 3 Pharmacology, All India Institute of Medical Sciences, Rishikesh, Rishikesh, IND; 4 Biochemistry, All India Institute of Medical Sciences, Rishikesh, Rishikesh, IND; 5 Psychology, All India Institute of Medical Sciences, Rishikesh, Rishikesh, IND

**Keywords:** copd, c-reactive protein, cytokines, exercise therapy, inflammatory biomarkers, interleukin-6 (il-6), pulmonary rehabilitation, systemic inflammation, tumor necrosis factor-alpha (tnf-α)

## Abstract

Introduction: Chronic obstructive pulmonary disease (COPD) is characterized by persistent airway inflammation, progressive airflow limitation, and frequent exacerbations that adversely affect functional capacity and clinical outcomes. Pulmonary rehabilitation (PR) is an established non-pharmacological intervention that improves exercise tolerance, symptom burden, and quality of life. However, its effects on systemic inflammation remain unclear.

Methods: This narrative review aimed to synthesize current evidence on the impact of exercise-based PR on systemic inflammatory markers in COPD. The review further outlines pulmonary rehabilitation and explores the mechanisms and clinical significance of systemic inflammation in COPD, including its impact on exacerbations, prognosis, and comorbidities, as well as PR’s influence on inflammatory pathways. A comprehensive literature search was conducted using PubMed, Scopus, and EMBASE databases. Studies published in English over the past 15 years involving human participants were considered. Included studies were randomized controlled trials and clinical studies evaluating the effect of PR or exercise-based interventions on systemic inflammatory biomarkers.

Results: PR programs were predominantly multicomponent, combining aerobic and resistance training with breathing exercises and education, typically delivered over 8-12 weeks. Evidence regarding their impact on systemic inflammation was inconsistent despite improvements in physiological and functional outcomes, along with exercise capacity, symptom control, and quality of life. Pulmonary rehabilitation may modulate systemic inflammation in COPD, but effects are variable and not consistently demonstrated. Its clinical benefits are more reliably reflected in functional and symptomatic improvements rather than biomarker changes. Further standardized, high-quality studies are needed to clarify the long-term anti-inflammatory potential of PR.

## Introduction and background

Chronic obstructive pulmonary disease (COPD) is a relentless respiratory disorder marked by persistent airway inflammation and fixed airway limitations. Its course is marked by frequent exacerbations that impact both the patients' functional capacity and outcomes [1]. Globally, COPD is a substantial contributor to mortality and is currently the fourth most prevalent cause of death, with an estimated prevalence of around 7.4% [2-4].

Pharmacological interventions, including bronchodilators, inhaled corticosteroids, and combination therapies, remain the cornerstone of COPD management and play a crucial role in symptom control and exacerbation prevention. However, these therapies may be associated with adverse effects such as dry mouth, tremors, oral candidiasis, and an increased risk of pneumonia, which may limit their long-term use in some patients [5]. Consequently, alternative and adjunctive non-pharmacological approaches have gained considerable attention for their potential to improve health outcomes in COPD. Pulmonary rehabilitation (PR) is increasingly recognized as an essential component of comprehensive COPD treatments along with standard pharmacological treatment [6]. It is a multidisciplinary and non-pharmacological approach that integrates structured exercise training and education for the patient with behavioral modification techniques. Various clinical trials showed that participation in pulmonary rehabilitation programs enhances exercise capacity and alleviates symptom burden and improves health-related quality of life, as well as decreases the risk of hospitalization [7]. Despite proven therapeutic advantages, the implementation of conventional pulmonary rehabilitation is hindered by practical challenges such as limited accessibility, economic constraints, and difficulties in maintaining adequate patient adherence [8]. To overcome such challenges, home-based or tele-rehabilitation approaches are being gradually implemented as feasible substitutes. It involves remote monitoring and specially designed exercise programs to achieve similar therapeutic outcomes and encourages patients to participate in their home setting [9]. The clinical benefit of both conventional and home-based or telepulmonary rehabilitation has been widely documented; however, its impact on systemic inflammatory pathways remains unexplored. Given that systemic inflammation plays a key role in disease progression and significantly impacts the development of comorbidities and overall prognosis, it is crucial to understand the impact of pulmonary rehabilitation on inflammatory markers [10]. This narrative review examines and synthesizes the existing evidence on the impact of exercise-based pulmonary rehabilitation on systemic inflammation in COPD and explores underlying biological mechanisms, drawing on findings from clinical studies and emerging perspectives.

## Review

Pulmonary rehabilitation: an overview

As COPD produces significant allostatic load on the respiratory system with time, holistic pulmonary rehabilitation (PR) is increasingly being accepted as a part of management aimed at improving quality of life far beyond just reducing mortality risk [1]. The American Thoracic Society (ATS) and European Thoracic Society (ERS) gave a joint statement in 2013, describing pulmonary rehabilitation as an evidence-based program built on detailed patient lifestyle assessment and utilizing effective non-pharmacological lifestyle modification interventions delivered through a multidisciplinary approach, customized to individual needs. The primary goal is to inculcate sustained adoption of healthy lifestyle practices monitored by the healthcare provider to enhance the overall physical and psychological well-being of individuals with chronic respiratory conditions [11]. Core elements of PR typically include structured exercise training targeted towards improving respiratory muscle strength, stamina, compliance, and ventilation-perfusion optimization; providing nutritional support to minimize inflammation; health education specific to the patient's disease status to improve independence in self-management; behavior modification therapy; and psychosocial counseling to improve the patient's perception of life satisfaction [12].

PR’s benefits in COPD patient care have already been well-traced through various research models. Consistent gains in the six-minute walk distance (6MWD) and enhanced peak oxygen uptake following PR establish improved exercise tolerance [13]. Betterment in respiratory symptoms, such as improved shortness of breath, is frequently reported, allowing patients to perform daily activities with greater ease [14]. Furthermore, PR has been shown to lower the frequency of hospital admissions, improve health-related quality of life, and reduce the severity of acute exacerbations, ultimately reducing healthcare burden and saving patients' costs [15]. Although PR was initially designed to relieve symptoms and improve the functional capacity of the respiratory system, it has proven to provide benefits beyond the immediate local disease, hence useful to address the systemic inflammatory pathophysiology, which is both caused by COPD and exacerbates its damage to the lungs, forming a vicious cycle, stagnating patient prognosis, and increasing mortality risk. Exercise training directly affects muscle strength, cardiovascular endurance, and metabolic regulation to combat systemic inflammation. Reduced psychological distress, better mental health, and behavioral strategies to cope with the long-term disease can also be implicated in this benefit [16]. This modulation of systemic inflammation in response to PR has been measured much more directly with reductions in biomarkers like CRP, IL-6, and TNF-α [17,18].

Systemic inflammation in chronic obstructive pulmonary disease (COPD)

Pathophysiology of Systemic Inflammation in COPD

COPD is characterized by persistent irritating stimuli (greater smoking pack years, inhabitants of high smog-laden areas, noxious gases from clay stoves, etc.) in the airway, leading to large and small airway inflammation. The EPI-SCAN study has provided evidence of significantly elevated levels of circulating inflammatory markers, such as IL-8, TNF-α, and NO metabolites, in COPD patients compared with healthy controls. Those same studies demonstrate an inverse correlation of these cytokines with lung function tests and exercise tolerance [19].

Cytokines like IL-6 or TNF-α cause the release of acute phase reactants from hepatocytes, such as CRP, fibrinogen, and serum amyloid A (SAA) protein. There is systemic recruitment of other cells, such as lymphocytes, neutrophils, mast cells, and eosinophils, causing exponential release of more inflammatory cytokines and sustained disordered activation of immunity over the years, causing extensive tissue injury throughout the body. This leads to endothelial dysfunction and vascular inflammation, leading to end-capillary tissue damage in vital organs, leading the body away from homeostasis and involving multiple systems dysregulation. It exaggerates airway and alveolar response to irritant stimuli, further worsening airflow obstruction and decreasing pulmonary ventilation. Meta-analyses have demonstrated the association of elevated blood levels of IL-6, CRP, ICAM-1, and SAA with disease progression and multisystem involvement in COPD, reinforcing the role of systemic inflammation [20].

Another meta-analysis showed significantly increased serum levels of TNF-α, IL-6, IL-8, CRP, and fibrinogen in COPD patients compared with controls, connecting local and systemic inflammation [21]. CRP and IL-6 in particular have emerged by prospective cohort studies to be independent indicators of disease severity and adverse clinical outcomes, even after adjustment for confounding factors. TNF-α contributes to systemic manifestations such as muscle wasting, while fibrinogen, an acute-phase reactant, reflects both inflammatory and pro-thrombotic states [22]. These findings support the concept that systemic inflammation is an intrinsic component of COPD pathophysiology as well as an indicator of disease severity.

Clinical Implications of Systemic Inflammation in COPD

Comorbidities: Comorbidities related to COPD are directly modulated by systemic inflammation. Large cohort syntheses show that CVS disorders, anemia of chronic inflammation, metabolic syndrome with its further complications like diabetes, dyslipidemia, and even depression, are found more prevalent and with greater severity in COPD patients with greater markers of systemic inflammation [23]. Recent data emphasize that comorbid conditions are associated with poorer clinical control, increased disease severity, and higher inflammatory burden, emphasizing the bidirectional relationship between systemic inflammation and multimorbidity [24]. Systemic inflammation appears to be modifiable and can be managed through various lifestyle modifications. Further systematic reviews and meta-analyses have shown that pulmonary rehabilitation leads to significant reductions in CRP, suggesting that non-pharmacological interventions can favorably influence systemic inflammatory status [25].

Exacerbations and prognosis: Elevated systemic inflammatory burden is strongly associated with increased exacerbation frequency and poorer long-term outcomes. Evidence demonstrates that higher levels of inflammatory markers predict exacerbations, hospitalization, and mortality, highlighting systemic inflammation as a prognostic indicator in COPD [26]. 

Biological basis of how PR may influence systemic inflammation: during exercise, skeletal muscle releases myokines that influence the immune system. Interleukin-6 (IL-6) is especially important, while chronically high IL-6 in COPD drives inflammation and acute-phase proteins like CRP and fibrinogen, the IL-6 released during exercise has the opposite effect. It helps trigger anti-inflammatory signals, boosting IL-10 and IL-1 receptor antagonists while dampening TNF-α. This pathway is thought to explain much of the anti-inflammatory benefit of regular training [26]. In COPD, pulmonary rehabilitation has been associated with reduced levels of indicators such as CRP and IL-8, as well as improved physical function. Still, results are unclear, as several studies indicate alterations in IL-6 or TNF-α, likely reflecting differences in exercise intensity, program length, and patient characteristics [27,28].

In addition, inflammation and oxidative stress are strongly linked; yet consistent training can help reestablish balance by enhancing mitochondrial function and improving antioxidant defenses. A single session of exercise may temporarily elevate oxidative stress; however, prolonged exercise programs reduce it by alleviating systemic inflammation and supporting efficient muscle metabolism [29].

PR improves a patient's ability to perform structured exercise and regular activities. Research indicates that exercise and home-based programs can help to reduce CRP and IL-8 levels while enhancing fitness and strength [27]. However, some studies find no cytokine changes after PR, indicating that outcomes may depend on disease severity, baseline inflammation, and exercise dose [30].

Individuals with COPD frequently experience mental health challenges, including stress, anxiety, sleep disturbances, and depression [22]. These factors contribute to increased systemic inflammation, mostly caused by dysregulation of the hypothalamic-pituitary-adrenal (HPA) axis and increased sympathetic nervous system activity. Engaging in exercise-based rehabilitation has demonstrated the capacity to improve psychological well-being while additionally lowering inflammatory indicators such as CRP, TNF-α, and IL-6 [31].

Methods

This narrative review was conducted between October 2025 and March 2026. A comprehensive literature search was performed on PubMed, Scopus, and EMBASE databases with the keywords (COPD), (pulmonary rehabilitation), (exercise therapy), (respiratory therapy), (systemic inflammation), (CRP), (interleukins), (TNF-α), (cytokines), (fibrinogen), (leukocytes), and (oxidative stress), combined using Boolean operators.

Additional manual searches were conducted using reference lists from relevant papers, and eligible articles were included in this review. Studies published between January 2010 and October 2025 were incorporated into the systematic narrative table. All relevant studies published in English and involving human participants were included. Priority was given to randomized controlled trials, clinical trials, and validation studies assessing the impact of pulmonary rehabilitation or exercise-based therapies on systemic inflammatory markers in individuals with COPD. The characteristics and key findings of the included studies related to exercise-based pulmonary rehabilitation and systemic inflammation in COPD are summarized in Table 1 [32-46,29].

**Table 1:** A Systematic Review Table for Exercise-Based Pulmonary Rehabilitation on Systemic Inflammation in COPD Age group data are presented as mean ± standard deviation, range, or as reported in the original studies. Intervention protocols, duration, intensity, and follow-up periods varied across studies. Where control or comparator groups were not applicable, “N/A” is indicated. COPD: chronic obstructive pulmonary disease; PR: pulmonary rehabilitation; RCT: randomized controlled trial; HRmax: maximum heart rate; Borg: Borg Rating of Perceived Exertion; 1-RM: one-repetition maximum; TBRS: total body recumbent stepper; TC: Tai Chi; TBRS–TC: combined total body recumbent stepper and Tai Chi; IMT: inspiratory muscle training; CAT: COPD assessment test; SF-36: 36-item Short Form Health Survey; SGRQ: St George’s Respiratory Questionnaire; WBC: white blood cells; ESR: erythrocyte sedimentation rate; SAA: serum amyloid A; NO: nitric oxide; N/A: not applicable. Inflammatory, immune, and oxidative stress markers: CRP, C-reactive protein; hs-CRP, high-sensitivity C-reactive protein; TNF-α, tumor necrosis factor-alpha; IFN-γ, interferon-gamma; IL, interleukin; TGF-β, transforming growth factor-beta; Th17, T helper 17 cells; Treg, regulatory T cells; CD, cluster of differentiation (including CD3+, CD4+, CD8+, CD11b, CD62L, CD163); TBARS, thiobarbituric acid reactive substances; apoB48, apolipoprotein B48; copeptin, C-terminal provasopressin marker; fibrinogen, plasma fibrinogen; leukocytes (including neutrophils, eosinophils, and lymphocytes).

Author and year	Country	Study Design	Gender	Age Group/mean age (interventional group)	Age Group/mean age Control group)	Sample Size	Intervention	Control	Duration of intervention	Frequency of Intervention (weeks)	Time (mins)	Mode of delivery (online/offline/home-based)	Outcome measures (in terms of systemic inflammation/systemic immune/muscle cytokines/inflammatory cell phenotypes/systemic oxidative stress/metabolic inflammatory markers)	Conclusion
Ito et al., 2025 [32].	Brazil	RCT	Both gender	67 (64.6–70.6)	67 (62.7–68.6)	Total= 50 25= Treadmill aerobic training 25= Usual care	Treadmill aerobic training (50–80% HRmax, progressive intensity, SpO₂ > 88%) and resistance training (biceps, deltoid, quadriceps, hamstrings; 3 sets, 8–12 repetitions, progressive load).	Usual care	8 weeks	3 times/week	1 hour	Offline/outpatient (pulmonary rehabilitation clinic)	TNF-α, IFN-γ, Th17 cells, Treg cells, IL-1β, IL-6, IL-17 and IL-10	Significant changes observed in Th17 inflammatory response, Treg cell phenotypes.
Muñoz Montiel A, et al., 2024 [33].	Spain	Prospective observational study	Both gender	64.5 year (64.5 ± 5.8)	N/A	Total=50 28=exacerbators, 22=non-exacerbators	Pulmonary rehabilitation program (PRP) included respiratory re-education (diaphragmatic/pursed-lip breathing, expiratory techniques, cough training, inspiratory muscle training), upper limb (dumbbells, arm ergometer) and lower limb training (cycling with progressive resistance). Intensity individualized (≈60–80% HRmax, Borg scale guided.	N/A	8 weeks	4 times/week	2 hours	Offline/program visit	fibrinogen, hs-CRP, TBARs, apoB48	No significant change in systemic inflammatory markers.
Valero-Breton M, et al., 2023 [34].	Chile	RCT	Both gender	71.1 ± 10.3	68.2 ± 10.0	Total=20 10=eccentric cycle training 10= concentric cycle training	Concentric Training (CONC): concentric cycling on recumbent ergometer, 2–3 sessions/week, 10–30 min, moderate intensity (RPE 11–13, Borg scale), progressive workload (~78 kJ average).	Eccentric Training (ECC): eccentric cycling on recumbent ergometer (resisting backward crank movement), 2–3 sessions/week, time duration 10–30 min, moderate intensity (RPE 11–13), higher workload (~227 kJ average).	12 weeks	2-3 times/week	10-30 min	Offline/out-patient	IL-6, TNF-α, IL-1β and thiobarbituric acid reactive substances (TBARS)	No significant change in systemic inflammatory markers.
Liu W, et al., 2023 [35].	China	RCT	Both gender	TC: 64.70 ± 6.89 TBRS: 65.47 ± 6.00 TBRS-TC: 63.13 ± 8.87	60.90 ± 7.44	Total=120/102 26=Comparator group 25=interventional group 26=Tai Chi group 25=TBRS-TC (interventional group)	(TC)Tai Chi group: Participants performed the 24-form Yang-style Tai Chi for 30 minutes per session, three sessions weekly, over an 8-week period. Mode: Supervised by certified Tai Chi instructors. TBRS group (total body recumbent stepper): Seated low-impact TBRS exercise, 30 min/session (including warm-up/cool-down), 3 times per week for 2 months. Mode: Supervised by physiotherapist. TBRS–TC group: Participants completed a combined program consisting of 15 minutes of TBRS exercise followed by 15 minutes of Tai Chi per session, conducted three times weekly for 8 weeks.	Routine medical care plus weekly patient education covering smoking cessation, medication management, exacerbation prevention, psychological support, nutrition, oxygen therapy, and breathing/cough training. Duration: 2 months. Frequency: Weekly education. Mode: Classroom/rehabilitation room instruction.	2 months to 12 months follow-up	3 times/week	30 min	Offline/out-patient (hospital based)	IL-6, Copeptin, IL-8 and TNF-α,	The combined total body recumbent stepper and Tai Chi (TBRSTC) intervention resulted in significantly greater reductions in IL-8 and TNF-α levels compared with the other study groups
Li Z, et al., 2023 [36].	China	RCT	Both gender	Stable group 64.40 ± 3.85 Acute group=64.22 ± 4.25	63.57 ± 4.12	Total=74 Stable group=58 Acute group=58 Control=58	comprehensive pulmonary rehabilitation (respiratory muscle training, pursed-lips and abdominal breathing, guided exercise, disease education, diet management, psychological counselling) and conventional drug therapy.	Conventional drug therapy (bronchodilators, ipratropium bromide, salbutamol, etc.) plus COPD education, diet guidance, and long-term medication per GOLD and NICE guidelines.	90 days	Not mentioned	Not mentioned	Not mentioned	TNF-α, IL-6 and hs-CRP	Both the acute and stable intervention groups exhibited significant reductions in systemic inflammatory markers, including TNF-α, hs-CRP, and IL-6, compared with the control group
Thyregod M, et al., 2022 [37].	Region Zealand, Denmark	Prospective, observational cohort study	Both gender	68 (range 50–80)	N/A	Total=57	Pulmonary rehabilitation included 1 h supervised exercise (strength training: 3×12 reps at 50–80% 1-RM for major muscles; endurance training: cycling/treadmill at moderate intensity, Borg 14–15) and 1 h patient education (disease, nutrition, pharmacology, smoking cessation).	N/A	7-12 weeks	2 times/week	2 hours	Offline/out-patient (rehabilitation centres or hospital based PR)	leukocytes, fibrinogen, serum CD163, eosinophils, CRP, IL 6, TNF α.	No significant change in systemic inflammatory markers.
Uzeloto et al., 2022 [38].	Brazil	RCT	Both gender	65 (65–72)	73.00 (67.25–77.25)	Total=23 15= Physical exercise group 8= Respiratory physiotherapy group	Physical exercise group(PEG): 3 sessions/week Intervention comprised dynamic warm-up and circuit training, 30-min treadmill aerobic exercise at ~80% 6MWT (Borg 4–6, progressive intensity), and upper- and lower-limb resistance training at 60–80% 1RM with gradual progression.	Respiratory physiotherapy group(RPG): 2 sessions/week Usual care involving inhaled therapy, deflation techniques, diaphragmatic exercises, and inspiratory muscle training.	8 weeks	2-3 times/week	30 min mentioned for aerobic exercises	Offline/out-patient	TNF-α, IL-6, IL-8, IL-17, IL-13, IL-2, and IL-10.	IL-8, TNF-α and IL-13 showed significant within-group changes, with no significant between-group differences.
Ma Y, et al., 2022 [39].	China	RCT	Both gender	68.64 (65.97–71.41)	70.70 (67.64–73.61)	Total=72 39= experimental group 33= control group	Same routine drug therapy and COPD education plus pulmonary rehabilitation training (pursed-lips breathing 5–10 min, 3–4 times/day; abdominal breathing 5–15 min, 3–4 times/day; skeletal muscle exercises 30 reps/set, 2–3 sets/day; relaxation training; coughing/expectoration techniques as needed).	Routine drug therapy (bronchodilator, glucocorticoid, expectorant, antitussive) plus COPD education (smoking cessation, healthy habits, diet).	12 week	2 times/week	40–60 minutes	Offline/out-patient (hospital based)	CD3+%, CD4+%, CD8+%, CD4+/CD8+%	CD3+%, CD4+%, and CD4+/CD8+% improved significantly, while CD8+% showed no significance difference.
Jenkins, at al., 2020 [40].	England (UK)	Prospective cohort study	Both gender	69 ± 7	N/A	Total=40 40= COPD phase 1 27= COPD phase 2	community pulmonary rehabilitation, 2 supervised sessions/week; each session 1 h aerobic exercises (shuttle walk, step-ups, timed up-and-go) and resistance exercises (biceps curls, wall press, lateral raise, cross-and-reach) at Borg intensity 4–5, plus 1-hour education; no nutritional intervention provided.	N/A	8 week	2 times/week	1hour exercise 1 hour education	Offline/out-patient	neutrophil activation markers CD11b, CD62L, CD66b, CD16bhigh/CD62Lhigh, CD16bhigh/CD62Llow, CD16blow/CD62Llow, CD16blow/CD62Lhigh, fibrinogen, CRP, total leukocytes, neutrophils, eosinophils, lymphocytes	Total leukocytes, neutrophils, immature neutrophil subset, and fibrinogen showed significant changes.
Wang J et al., 2020 [41].	China	RCT	Both gender	65.93±7.87	66.65±6.99	Total=150 80=observational group 75= control group	Conventional treatment plus individualized cardiopulmonary rehabilitation therapy for 1 month, including exercise (twice daily, 1 h each), breathing training (three times weekly, 15 min each), and ongoing psychological counselling, all guided by rehabilitation physicians.	Conventional treatment only	1 month	3 times/week	1 hour	Offline/out-patient	TNF-α, CRP, IL-10, and TGF-β	The rehabilitation group showed increased IL-10 and TGF-β and decreased CRP and TNF-α relative to controls.
Neunhäuserer, et al., 2020 [42].	Austria	RCT	Both gender	63.5 (± 5.9)	N/A	Total=29	Combined endurance and strength training, Sessions included 5-min warm-up, 7×1-min high-intensity cycling intervals (70–80% peak work rate) with 2-min active recovery, 5-min cool down, plus 8 high-intensity strength exercises; intensity progressively adapted.	Cross over of breathing medical air or supplemental oxygen	12 week (crossover after 6 week_	3 times/week	45-50 min	Offline/out-patient	leptin, TNF α, IL 6, hsCRP, fibrinogen, total leukocytes, eosinophils	Significant reductions were observed in fibrinogen, leptin, eosinophils, and late EPCs.
Ryrsø, et al., 2018 [43].	Denmark	RCT	Not mentioned	63 ± 2	64 ± 3	Total=38 15= resistance training 15= exercise training 8= healthy control	ET group: 35 min supervised aerobic training (ergometer cycling/treadmill walking, Borg 14–15), RT group: 35 min supervised resistance training (chest press, rowing, leg press, leg extension; 4 sets, 30 s each, 30–40% 1RM)	Healthy control	8 weeks	3 times/week	35 min	Offline/out-patient	Immune cell counts; serum CRP; plasma IL-1β, IL-6, IL-8, IL-18, TNF-α; muscle CD163; M1/M2 ratio; muscle IL-1β, IL-6, IL-8, IL-18, TNF-α	No significant changes were observed in systemic inflammatory markers.
Sciriha et al., 2017 [44].	Malta	Longitudinal observational study	Both gender	66 ± 7.76	N/A	49	Pulmonary rehabilitation, included Warm-up; treadmill (progressive per 6MWT); stair climbing; arm ergometry; cycling; limb strengthening (weights); IMT (Respironics IMT Threshold, 15 + 30 min, 5 days/week).	N/A	12 week	2 times/week	2 hours	Home-based	complete blood count, ESR, serum CRP, serum SAA, exhaled nitric oxide (NO)	No significant change in systemic inflammatory markers.
Abd El-Kader et al., 2016 [45].	Saudi Arabia	Comparative study	Both gender	34.17± 6.25	36.14± 4.79	Total = 80/100 40=aerobic exercises 40= resistance exercises	Aerobic exercise: Aerobic treadmill training, 3×/week for 3 months (5-min warm-up, 30-min aerobic at 60–70% HRmax, 5-min cool-down).	Resistance exercise: Resistance training, (~60 min/session, 70–85% 1-RM, major and minor muscle groups, 3 sets of 15 reps for abs and calf). Both supervised by physical therapists.	12 weeks	3 times/week	40 min for aerobic exercise 60 min for resistance exercise	Offline/out-patient	IL-2, TNF α, CRP, IL-4 and IL-6	Both groups showed significant decreases in IL-2, TNF α, CRP, IL-4 and IL-6 with superior reductions in the aerobic exercise group.
do Nascimento ES et.al.,2015 [29].	Brazil	Longitudinal observational study	Both gender	64.8±5.1	N/A	14	Patients received an exercise booklet with disease education and were instructed to perform a home-based routine (warm-up, aerobic walking, resistance, stretching, relaxation) 3 times per week for 8 weeks. Progress was monitored with diaries and fortnightly visits for adjustments.	N/A	8 week	3 times / week	Only 40 min mentioned for aerobic exercises	Home- Based	IL-6, IL-8	No significant change in systemic inflammatory markers.
Greulich et al., 2014 [46].	Germany	RCT	Both gender	64.61 ± 9.02	65.83 ± 8.59	Total=61 34 included in final measurement	Individualized Training: Weekly individualized gym-based program per ACCP/AACVPR guidelines, tailored to each patient’s maximal force and endurance.	Non-Individualized Training: Weekly group calisthenics: 10 min warm-up, 40 min collective exercises (ball games, stepping, thera-band, dumbbells), 10 min relaxation.	3 months	1 time/week	60 min	Offline (gym-based/group calisthenics )	WBC, TNF α, IL 8, CRP, IL 6,	No significant changes were observed in systemic inflammatory markers.

Discussion

Pulmonary rehabilitation interventions reported in the literature are mostly exercise-centered, multicomponent programs combining aerobic endurance training with resistance exercises for major upper- and lower-limb muscle groups. It is also complemented by breathing retraining techniques and structured educational sessions. Aerobic exercise is typically prescribed at moderate-to-high intensity (approximately 60-80% of HRmax or Borg 4-6/14-15), with intensity guided by heart rate targets or perceived exertion scales. On the other hand, resistance training typically employs progressive loading (50-80% 1RM) based on one-repetition maximum or functional capacity. Less commonly studied interventions include eccentric cycling, high-intensity interval training, and the integration of other interventions with PR, such as Tai Chi, functional circuit-based exercise, and seated recumbent stepper programs, which are generally evaluated in trials.

Most programs are delivered over 8-12 weeks, with supervised sessions held 2-3 times a week and lasting 30-120 minutes, depending on whether educational and respiratory components are incorporated. Delivery is predominantly hospital- or rehabilitation center-based, although community- and home-based formats are occasionally used. Comparator conditions vary substantially and include usual care, standard pharmacological management with education, respiratory physiotherapy alone, alternative or lower-intensity exercise regimens, healthy control groups, and crossover designs involving adjuncts such as supplemental oxygen. This highlights the methodological heterogeneity of pulmonary rehabilitation research [32-46,29].

Pulmonary Rehabilitation and Systemic Inflammatory Markers

Findings regarding the influence of pulmonary rehabilitation on systemic inflammatory markers in COPD remain inconsistent, with only some studies demonstrating meaningful anti-inflammatory effects. Several randomized and other clinical trials evaluating structured aerobic or multicomponent rehabilitation have reported a significant decrease in circulating pro-inflammatory biomarkers, such as TNF-α, IL-6, and CRP, together with increases in anti-inflammatory cytokines, including IL-10 and TGF-β, compared with usual or standard care [45,36,41].

Similarly, a randomized crossover trial program combining endurance and resistance exercises was delivered with or without supplemental oxygen. It resulted in significant reductions in fibrinogen and leptin levels, along with beneficial changes in eosinophil counts among patients with endothelial dysfunction. These findings indicate a potential systemic anti-inflammatory effect associated with higher-intensity training [42]. Improvements in inflammatory balance were also observed at the cellular level, where combined aerobic and resistance training significantly modulated Th17/Treg cell profiles and immunosuppressive activity, indicating an exercise-induced shift toward an anti-inflammatory immune phenotype [32].

In contrast, findings from several well-conducted observational and randomized studies indicate traditional systemic inflammatory biomarkers remained unchanged, despite evident gains in functional capacity following pulmonary rehabilitation [44,43,37]. Studies evaluating standard outpatient or community-based rehabilitation programs frequently reported no significant changes in CRP, IL-6, TNF-α, fibrinogen, or leukocyte subpopulations following the intervention, despite improvements in exercise performance and health-related quality of life [33,40,29]. Similarly, trials comparing aerobic versus resistance-only training or concentric versus eccentric cycling did not observe differential effects on systemic cytokines, suggesting that exercise modality alone may be insufficient to elicit measurable inflammatory changes over short intervention periods [34,43]. Collectively, the available evidence suggests that pulmonary rehabilitation does not uniformly produce anti-inflammatory effects; rather, its impact appears to vary according to baseline inflammatory burden, training intensity, length of intervention, and the extent of individualized, multimodal program elements.

Significant Physiological, Functional, and Immunological Outcomes

Beyond systemic inflammatory markers, pulmonary rehabilitation consistently demonstrated robust improvements in functional capacity, muscle strength, metabolic health, and immune regulation across multiple study designs. Significant improvements in exercise capacity, as measured by increases in six-minute walk distance and peak work rate, were consistently reported after multicomponent rehabilitation programs, even in studies that showed no significant changes in inflammatory markers [32,44,37]. Notable enhancements in peripheral muscle strength and overall physical fitness in patients were also observed, especially in programs that integrated aerobic and resistance training with progressive overload [32,38,45]. Several studies also documented beneficial changes in metabolic and cardiovascular risk parameters, including reductions in glucose levels, HbA1c, BODE index, leptin concentrations, and cardiovascular risk scores, indicating systemic effects extending beyond pulmonary function [33,42]. Pulmonary rehabilitation has also been shown to produce measurable immunological changes beyond traditional inflammatory markers in individuals with stable COPD. Some studies reported improvements in T-cell profiles, including increased CD3⁺ and CD4⁺ counts and higher ratios of CD4⁺/CD8⁺, following comprehensive rehabilitation programs [37]. Exercise-related alterations in cytokine expression within immune cells were also observed by changes in IL-8, IL-13, and TNF-α production in CD4⁺ T lymphocytes. However, these effects were not consistently different when compared with control groups [38]. Novel exercise modalities like Tai Chi and total-body recumbent stepping have been associated with significant reductions in IL-8 and TNF-α, suggesting that mind-body and hybrid training strategies may contribute to systemic immunomodulation [35]. Collectively, the evidence indicates that pulmonary rehabilitation confers extensive multisystem benefits.

Functional Exercise Capacity and Physical Performance

Improved functional exercise performance represents one of the most consistently observed outcomes of pulmonary rehabilitation. Multiple trials have demonstrated significant gains in exercise tolerance, commonly evaluated through the 6-minute walk test, peak work rate, or maximal oxygen consumption [44,46,41,35,36]. Programs combining endurance and strength training resulted in significant gains in 6MWT, peripheral muscle strength, and daily activity levels, reflecting broad functional recovery rather than isolated performance benefits [32]. Similarly, high-intensity endurance and resistance training protocols significantly increased peak work rate and VO₂, reflecting improved cardiopulmonary efficiency [42]. Importantly, clinically meaningful functional improvements were also observed in non-hospital settings. Home-based rehabilitation improved exercise tolerance and inspiratory muscle strength, supporting its role as a feasible alternative where access to center-based programs is limited [29]. Multidisciplinary outpatient rehabilitation also demonstrated improvements in composite prognostic indices, such as the BODE index, and in metabolic parameters in patients who achieved meaningful improvements in walking distance [33]. Long-term benefits were particularly evident in programs combining traditional exercise with mind-body components, where combined Tai Chi and total-body recumbent stepping maintained functional improvements up to 12 months [35].

Several studies demonstrated improvements in objective physiological measures following structured pulmonary rehabilitation. Significant improvements in spirometric parameters, including FEV1 and FEV1/FVC ratio, were observed in comprehensive rehabilitation programs integrating respiratory training, exercise therapy, and education. These improvements were accompanied by enhanced daily activity performance and reduced symptom burden, also when rehabilitation was delivered as part of multidisciplinary care [36,39,41]. Beyond pulmonary mechanics, rehabilitation also improved broader cardiopulmonary and systemic physiological performance. High-intensity interval training was associated with enhanced oxygen utilization efficiency and improved cardiovascular responses during exercise in patients [42]. In addition, comprehensive rehabilitation programs that incorporated breathing techniques, structured physical training, and behavioral strategies led to improvements in pulmonary function, alongside reductions in exacerbation frequency and symptom burden [35].

Quality of Life and Symptom Control

Enhanced quality of life was consistently observed across diverse comprehensive rehabilitation programs. Structured interventions that integrate exercise training with educational, behavioral, and psychological components produced meaningful reductions in disease impact and symptom burden, along with significant improvements in health-related quality-of-life measures [32,41,39,35,36,29]. These benefits were frequently associated with reduced dyspnea severity, perceived fatigue, and psychological distress. However, improvements in quality-of-life outcomes were not consistently demonstrated across all pulmonary rehabilitation formats. For instance, individualized gym-based training enhanced muscle morphology and walking performance, but it did not yield significant changes in patient-reported quality-of-life measures when compared with group-based exercise programs [46].

Clinical Implications of PR on Systemic Inflammatory Markers

A number of investigations have examined whether PR influences systemic inflammatory biomarkers, including CRP, IL-6, and TNF-α. However, the results remain inconclusive. A 2024 meta-analysis indicated a potential increase in systemic inflammatory markers following pulmonary rehabilitation. However, this observation was mainly influenced by a single study and lost significance after sensitivity analysis, reflecting the variability and limited strength of the evidence. In contrast, a 2025 systematic review indicated that PR programs of at least four weeks’ duration may be associated with reductions in CRP levels, while consistent effects on IL-6 and fibrinogen were not demonstrated. Taken together, the overall certainty of the evidence is low [25,47]. From a clinical standpoint, PR should not be presumed to uniformly reduce systemic inflammation. Transient increases in inflammatory biomarkers observed after exercise may reflect a physiological adaptive response rather than an adverse outcome. On the other hand, the benefits of pulmonary rehabilitation are consistently reflected in enhanced exercise capacity, reduced symptom severity, and significant gains in health-related quality of life. These functional and symptomatic gains are more consistently demonstrated than changes in circulating inflammatory markers. PR should therefore be recommended for its proven clinical effects, with further research needed to determine its long-term anti-inflammatory impact.

## Conclusions

Evidence suggests that pulmonary rehabilitation can influence systemic inflammatory processes in COPD, but these effects are inconsistent and appear to be influenced by factors such as training intensity, program duration, and overall program design. While some well-designed interventions show reductions in pro-inflammatory markers, some demonstrate only limited biomarker changes despite consistent improvements in functional, metabolic, and immunological outcomes. Collectively, this evidence supports pulmonary rehabilitation as an effective multisystem therapy while emphasizing the need for protocol standardization and focused research to better define its role in modulating systemic inflammation. 

This review has some limitations. As a narrative review, its primary objective was to examine and synthesize the existing evidence on the impact of exercise-based PR on systemic inflammation and to explore the underlying mechanisms and the clinical significance of systemic inflammation in COPD. Consequently, a quantitative meta-analysis was not performed, and formal risk-of-bias and certainty-of-evidence assessments were not undertaken. Therefore, the findings should be interpreted as a qualitative synthesis of the available literature.

## References

[1] GOLD Report and Pocket Guide (2026). Global Initiative for Chronic Obstructive Lung Disease: 2026 GOLD report and pocket guide. https://goldcopd.org/2026-gold-report-and-pocket-guide/.

[2] (2026). World Health Organization: Chronic obstructive pulmonary disease (COPD). https://www.who.int/news-room/fact-sheets/detail/chronic-obstructive-pulmonary-disease-(copd).

[3] Daniel RA, Aggarwal P, Kalaivani M, Gupta SK (2021). Prevalence of chronic obstructive pulmonary disease in India: A systematic review and meta-analysis. Lung India.

[4] Jo YS (2022). Long-term outcome of chronic obstructive pulmonary disease: a review. Tuberc Respir Dis (Seoul).

[5] Duan R, Li B, Yang T (2023). Pharmacological therapy for stable chronic obstructive pulmonary disease. Chronic Dis Transl Med.

[6] Safka KA, McIvor RA (2015). Non-pharmacological management of chronic obstructive pulmonary disease. Ulster Med J.

[7] Lu HY, Chen CF, Lee DL (2023). Effects of early pulmonary rehabilitation on hospitalized patients with acute exacerbation of chronic obstructive pulmonary disease: a systematic review and meta-analysis. Int J Chron Obstruct Pulmon Dis.

[8] Casaburi R, ZuWallack R (2009). Pulmonary rehabilitation for management of chronic obstructive pulmonary disease. N Engl J Med.

[9] Reychler G, Piraux E, Beaumont M (2022). Telerehabilitation as a form of pulmonary rehabilitation in chronic lung disease: a systematic review. Healthcare (Basel).

[10] Singh S, Verma SK, Kumar S (2018). Correlation of severity of chronic obstructive pulmonary disease with potential biomarkers. Immunol Lett.

[11] Spruit MA, Singh SJ, Garvey C (2013). An official American Thoracic Society/European Respiratory Society statement: key concepts and advances in pulmonary rehabilitation. Am J Respir Crit Care Med.

[12] Nici L, Donner C, Wouters E (2006). American Thoracic Society/European Respiratory Society statement on pulmonary rehabilitation. Am J Respir Crit Care Med.

[13] Moore LE, Byers BW, Fuhr DP (2017). Cardiovascular benefits from standard pulmonary rehabilitation are related to baseline exercise tolerance levels in chronic obstructive pulmonary disease. Respir Med.

[14] Osadnik CR, Loeckx M, Louvaris Z (2018). The likelihood of improving physical activity after pulmonary rehabilitation is increased in patients with COPD who have better exercise tolerance. Int J Chron Obstruct Pulmon Dis.

[15] Puhan M, Scharplatz M, Troosters T (2009). Pulmonary rehabilitation following exacerbations of chronic obstructive pulmonary disease. Cochrane Database Syst Rev.

[16] McCarthy B, Casey D, Devane D (2015). Pulmonary rehabilitation for chronic obstructive pulmonary disease. Cochrane Database Syst Rev.

[17] Garrod R, Ansley P, Canavan J, Jewell A (2007). Exercise and the inflammatory response in chronic obstructive pulmonary disease (COPD)--Does training confer anti-inflammatory properties in COPD?. Med Hypotheses.

[18] Xiong T, Bai X, Wei X (2023). Exercise rehabilitation and chronic respiratory diseases: effects, mechanisms, and therapeutic benefits. Int J Chron Obstruct Pulmon Dis.

[19] Garcia-Rio F, Miravitlles M, Soriano JB (2010). Systemic inflammation in chronic obstructive pulmonary disease: a population-based study. Respir Res.

[20] Li XF, Wan CQ, Mao YM (2022). Analysis of pathogenesis and drug treatment of chronic obstructive pulmonary disease complicated with cardiovascular disease. Front Med (Lausanne).

[21] Gan WQ, Man SF, Senthilselvan A, Sin DD (2004). Association between chronic obstructive pulmonary disease and systemic inflammation: a systematic review and a meta-analysis. Thorax.

[22] Strollo HC, Nouraie SM, Hoth KF (2021). Association of systemic inflammation with depressive symptoms in individuals with COPD. Int J Chron Obstruct Pulmon Dis.

[23] Putcha N, Drummond MB, Wise RA, Hansel NN (2015). Comorbidities and chronic obstructive pulmonary disease: prevalence, influence on outcomes, and management. Semin Respir Crit Care Med.

[24] Almagro P, Soler-Cataluña JJ, Huerta A (2024). Impact of comorbidities in COPD clinical control criteria. The CLAVE study. BMC Pulm Med.

[25] Farley C, Le Bouedec M, Oliveira A (2025). The effects of pulmonary rehabilitation on inflammatory biomarkers in patients with chronic obstructive pulmonary disease: a systematic review and meta-analysis. Can Jr Resp Cri Car Sle Med.

[26] Petersen AM, Pedersen BK (2006). The role of IL-6 in mediating the anti-inflammatory effects of exercise. J Physiol Pharmacol.

[27] Wang CH, Chou PC, Joa WC (2014). Mobile-phone-based home exercise training program decreases systemic inflammation in COPD: a pilot study. BMC Pulm Med.

[28] Newman AN, Oliveira A, Goldstein R (2023). The effects of pulmonary rehabilitation on inflammatory biomarkers in patients with chronic obstructive pulmonary disease: Protocol for a systematic review and meta-analysis. PLoS One.

[29] do Nascimento ES, Sampaio LM, Peixoto-Souza FS (2015). Home-based pulmonary rehabilitation improves clinical features and systemic inflammation in chronic obstructive pulmonary disease patients. Int J Chron Obstruct Pulmon Dis.

[30] Canavan J, Garrod R, Marshall J (2007). Measurement of the acute inflammatory response to walking exercise in COPD: effects of pulmonary rehabilitation. Int J Chron Obstruct Pulmon Dis.

[31] Abd El-Kader SM, Al-Jiffri OH (2016). Exercise alleviates depression related systemic inflammation in chronic obstructive pulmonary disease patients. Afr Health Sci.

[32] Ito JT, Alves LH, Oliveira LM (2025). Effect of exercise training on modulating the TH17/TREG imbalance in individuals with severe COPD: A randomized controlled trial. Pulmonology.

[33] Muñoz Montiel A, Ruiz-Esteban P, Doménech Del Río A (2024). The effect of pulmonary rehabilitation on cardiovascular risk, oxidative stress and systemic inflammation in patients with COPD. Respir Med.

[34] Valero-Breton M, Valladares-Ide D, Álvarez C (2023). Changes in blood markers of oxidative stress, inflammation and cardiometabolic patients with COPD after eccentric and concentric cycling training. Nutrients.

[35] Liu W, Liu XM, Huang YL (2023). Tai Chi as a complementary exercise for pulmonary rehabilitation in chronic obstructive pulmonary disease: A randomised controlled trial. Complement Ther Med.

[36] Li Z, Yan X, Liu J (2023). Value of comprehensive rehabilitation therapy in patients with chronic obstructive pulmonary disease and its effect on improving inflammation. Altern Ther Health Med.

[37] Thyregod M, Løkke A, Skou ST (2022). Changes in systemic inflammation after pulmonary rehabilitation in patients with COPD and severe physical inactivity - an exploratory study. Chron Respir Dis.

[38] Uzeloto JS, de Toledo-Arruda AC, Silva BS (2022). Effect of physical training on cytokine expression in CD4+ T lymphocytes in subjects with stable COPD. Ther Adv Respir Dis.

[39] Ma Y, Chen Y, Zhang N (2022). Efficacy and safety of pulmonary rehabilitation training on lung function, quality of life, and T cell immune function in patients with stable chronic obstructive pulmonary disease: a randomized controlled trial. Ann Palliat Med.

[40] Jenkins AR, Holden NS, Jones AW (2020). Inflammatory responses to acute exercise during pulmonary rehabilitation in patients with COPD. Eur J Appl Physiol.

[41] Wang J, Zhang Y, Zhou X (2020). Effects of rehabilitation training on cardiopulmonary function, life quality and inflammatory factors in serum of elderly patients with COPD. Int J Clin Exp Med.

[42] Neunhäuserer D, Patti A, Niederseer D (2021). Systemic inflammation, vascular function, and endothelial progenitor cells after an exercise training intervention in COPD. Am J Med.

[43] Ryrsø CK, Thaning P, Siebenmann C (2018). Effect of endurance versus resistance training on local muscle and systemic inflammation and oxidative stress in COPD. Scand J Med Sci Sports.

[44] Sciriha A, Lungaro-Mifsud S, Bonello A (2017). Systemic inflammation in COPD is not influenced by pulmonary rehabilitation. Eur Jr Phys.

[45] Abd El-Kader SM, Al-Jiffri OH, Al-Shreef FM (2016). Plasma inflammatory biomarkers response to aerobic versus resisted exercise training for chronic obstructive pulmonary disease patients. Afr Health Sci.

[46] Greulich T, Kehr K, Nell C (2014). A randomized clinical trial to assess the influence of a three months training program (gym-based individualized vs. calisthenics-based non-invidualized) in COPD-patients. Respir Res.

[47] Yue XA, Sheng Y, Li J (2024). Effects of pulmonary rehabilitation on systemic inflammation in chronic obstructive pulmonary disease: a meta-analysis. Am J Clin Exp Immunol.

